# Development of measures assessing attitudes toward contraband tobacco among a web-based sample of smokers

**DOI:** 10.1186/s12971-015-0032-0

**Published:** 2015-03-27

**Authors:** Sarah E Adkison, Richard J O’Connor, Michael Chaiton, Robert Schwartz

**Affiliations:** Department of Health Behavior, Roswell Park Cancer Institute, 60 Carlton Street, Buffalo, NY 14263 USA; Dalla Lana School of Public Health, University of Toronto, T523, 33 Russell St., Toronto, ON M5S 2S1 Canada; The Ontario Tobacco Research Unit, Dalla Lana School of Public Health, University of Toronto, 155 College Street, 5th Floor, Toronto, ON M5T3M7 Canada

**Keywords:** Structural equation modeling, Tobacco, Contraband tobacco, Public policy, Behavioral economics, Survey methods

## Abstract

**Background:**

As regulation of tobacco products tightens, there are concerns that illicit markets may develop to supply restricted products. However, there are few validated measures to assess attitudes or purchase intentions toward contraband tobacco (CT). As such, it is important to investigate individual level characteristics that are associated with the purchase and use of contraband tobacco.

**Methods:**

In May 2013, a pilot survey assessed attitudes, behaviors, and purchase intentions for contraband tobacco based on previous research regarding non-tobacco contraband. The survey was administered via Amazon Mechanical Turk, a crowdsourcing resource, among current smoking respondents in the United States and Canada. Structural equation modeling was used to evaluate the validity of the proposed model for understanding attitudes toward contraband tobacco.

**Results:**

CT purchasers were more likely to report norms supportive of counterfeit products, more intentions toward purchasing counterfeit products, a lowered risk associated with these products, and to have more favorable attitudes toward CT than those who had not purchased CT. Attitudes toward CT mediated the relationship between subjective norms and prior purchase with behavior intentions. Perceived risk had a significant direct effect on intentions and an indirect effect through attitudes toward CT. The structural model fit the data well and accounted for over half (53%) of the variance in attitudes toward tobacco.

**Conclusions:**

Understanding the mechanisms associated with CT attitudes and purchase behaviors may provide insight for how to mitigate possible iatrogenic consequences of newly implemented regulations. The measures developed here elucidate some elements that influence attitudes and purchase intentions for CT and may inform policy efforts to curtail the development of illicit markets.

**Electronic supplementary material:**

The online version of this article (doi:10.1186/s12971-015-0032-0) contains supplementary material, which is available to authorized users.

## Introduction

As taxes on tobacco products have increased, tax avoidance behaviors among smokers may also increase [1-5] and larger scale tax evasion schemes such as smuggling may become more prevalent. Thus, an international protocol to control the illicit trade of tobacco, focusing on the supply chain, has been negotiated as part of the Framework Convention on Tobacco Control (http://www.who.int/fctc/protocol/about/en/). However, taxes may not be the sole influence on contraband tobacco use. As regulation of tobacco products tightens, concerns have also been expressed that illicit markets may develop to supply restricted products (e.g., menthol cigarettes) [6,7]. Therefore, it is important to generate knowledge about what characteristics are associated with people who purchase and use contraband tobacco products in order to mitigate possible iatrogenic consequences of newly implemented regulations. For purposes of the current article, contraband tobacco is defined as tobacco that has been obtained outside the regulated supply chain, purchased without appropriate taxation, and/or tobacco sought out to avoid local or state taxes (e.g. purchase on an Indian reservation among non-natives).

### Background and conceptual model

While the scientific literature on contraband tobacco has examined prevalence of, attitudes toward, and correlates of contraband tobacco use [8-11], this literature has been limited by a dearth of validated measures [12]. Previous literature seeking to understand purchase intentions for contraband tobacco outline some economic indicators. These include how perceived product quality and price are associated with purchase intentions; [13,14] however, research has not evaluated how social elements may also contribute to attitudes toward contraband tobacco and purchase intentions. Greater understanding of these influences is needed. Some non-tobacco contraband/counterfeit literature examines personal and interpersonal factors that promote or inhibit attitudes toward these products that could be applied to the contraband tobacco issue. Examples of these non-tobacco contraband/counterfeit products include pirated cd’s, handbags, and pharmaceuticals.

**Figure 1:**
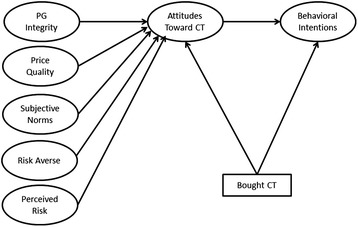
**Conceptual model (based on Augusto de Matos et al. (2007)** [16]**.**

Drawing on the literature regarding attitudes and behavioral intentions to purchase and use non-tobacco contraband/counterfeit products, the current research adapts a conceptual model (Figure 1) put forth by Augusto de Matos and colleagues in the context of counterfeit goods to explain behavioral intentions and attitudes toward contraband tobacco. Under the general framework of the Theory of Reasoned Action and Planned Behavior (TRA), this research highlights two primary antecedents of consumer attitudes and behaviors: Product demand (price and risk factors) and social components (social norms and personality factors).

### Product demand

While literature examining the psychosocial factors related to purchasing contraband tobacco is sparse, the broader literature on non-tobacco contraband and counterfeit products highlights a number of factors that influence product demand, including affordability/relative price and perceived quality. Affordability of non-tobacco contraband/counterfeit has been shown to increase consumer purchase intentions [15]. However, research also suggests that consumers associate lower price with a low quality or low performing product which deters purchase intentions [16,17]. These consumers are considered “risk averse” or have the “propensity to avoid taking risks (p.37)” [16]. Understanding how consumers interpret the price differential and product performance may effectively differentiate between consumers who have favorable or unfavorable attitudes toward contraband tobacco which may, in turn, influence future purchase intentions. According to TRA, favorable attitudes are influential in guiding future behavior [18]. We adopt measures from the non-tobacco contraband/counterfeit research to assess consumers price quality inference [19], perceived risk [15,20], and risk averseness [15], and their relation with contraband tobacco attitudes.

**H1:** Consumers who perceive that a higher priced product is associated with a better quality product (price quality) will have less favorable attitudes toward contraband tobacco and these attitudes will mediate the relationship between price quality and behavioral intentions.

**H2:** Consumers who perceive that the risk (perceived risk) associated with counterfeit/contraband products, in general, is high will have less favorable attitudes toward contraband tobacco and these attitudes will mediate the relationship between perceived risk and behavioral intentions

**H3:** Consumers who are prefer to avoid taking risks (risk averseness) when making purchases will have less favorable attitudes toward contraband tobacco and these attitudes will mediate the relationship between risk averseness and behavioral intentions.

**Table 1:** **Demographic characteristics by contraband tobacco purchase**

		**Non-buyers %**	**Buyers %**	**N (% sample)**	***x*** ^**2**^
Sex	Male	51.4	48.6	245 (50.7)	p < .000
	Female	70.2	29.8	238 (49.3)	
Race	White	63.0	37.0	357 (73.9)	p < .031
	Black	55.2	44.8	29 (6.0)	
	NA/FN	70.0	30.0	10 (2.1)	
	Asian	41.8	58.2	55 (11.4)	
	Multiracial/Other	68.8	31.3	32 (6.6)	
Hispanic	Yes	43.3	56.7	60 (12.4)	p = .003
	No	56.7	36.9	423 (87.6)	
Age	18-25	52.8	47.2	125 (25.9)	p = .223
	26-30	63.6	36.4	129 (26.7)	
	31-40	63.0	37.0	119 (24.6)	
	40+	63.6	36.4	110 (22.8)	
SDS	Low	60.7	39.3	61 (12.6)	p = .128
	Medium	57.2	42.8	278 (57.6)	
	High	67.4	32.6	144 (29.8)	

Note: NA/FN were not classified as “buyers” if they reported purchasing on an Indian Reservation or First Nations Reserve.

### Social norms and personality factors

The literature on non-tobacco contraband/counterfeits also highlights social and personality factors that influence both attitudes and purchase intentions, including personal ethical beliefs and subjective normative beliefs. The research on ethical beliefs indicates that consumers who value honesty and high self-control are less likely to hold favorable attitudes toward contraband products as they are more likely to feel a sense of guilt associated with the purchase behavior [21]. In addition, those who have previously purchased contraband or have significant others (close friends or family) who purchase or support the purchase of contraband products will experience social pressure to have more favorable attitudes toward contraband and purchase behavior. We utilize the measures from the non-tobacco contraband research to assess how integrity, personal gratification, and subjective norms, may be associated with attitudes toward contraband tobacco (Table 1) and behavioral intentions to purchase contraband products.

**H4:** Consumers who have high levels of integrity will have less favorable attitudes toward contraband tobacco and these attitudes will mediate the relationship between integrity and behavioral intentions

**H5:** Consumers who value personal gratification will have less favorable attitudes toward contraband tobacco and these attitudes will mediate the relationship between personal gratification and behavioral intentions

**H6:** Consumers who perceive their significant others (subjective norms) would approve of the behavior will have more favorable attitudes toward contraband tobacco and these attitudes will mediate the relationship between subjective norms and behavioral intentions

**H7:** Having previously purchased contraband tobacco will be associated with more favorable attitudes toward contraband tobacco and behavioral intentions.

The TRA and prior research on non-tobacco contraband consistently shows that product-related attitudes predict future purchase intention [18,22]. In other words, consumers who hold favorable attitudes toward a product are more likely to express behavioral intentions toward purchasing that product.

**H8:** Consumers with positive attitudes about contraband tobacco will have more favorable attitudes toward contraband tobacco higher behavioral intentions to purchase contraband products.

## Methods

In May 2013, a pilot survey was developed to assess attitudes, behaviors, and purchase intentions for contraband tobacco based on previous research regarding non-tobacco contraband [16]. The survey was administered using the Qualtrics web survey platform via Amazon Mechanical Turk (MTurk), a crowdsourcing resource. Crowdsourcing refers to the outsourcing of tasks to a large pool of individuals over the Internet in return for compensation and has been utilized (MTurk in particular) for a variety of academic social science research, where it has been shown to be a reliable and useful approach to data collection [23]. Furthermore, because of the large and diverse pool of MTurk workers, data is generated among a diverse sample [24-26] in a fast [27], inexpensive [25], and reliable way [24,28].

The study sample was limited to MTurk workers who were current smokers age 18 and older and lived in either the United States or Canada. Respondents first completed an informed consent and were then administered screening questions to assess whether they were qualified to complete the task. Smoking status was determined by a single question asking if the participant had smoked one or more cigarettes in the past 30 days. Respondents were compensated $1 USD for completing the 20 minute survey. This study was approved by the Institutional Review Board at Roswell Park Cancer Institute.

### Measures

#### Contraband tobacco

For this research, contraband tobacco is defined as tobacco obtained outside regulated wholesale and retail channels, bought without the requisite taxes applied (http://www.rcmp-grc.gc.ca/pubs/tobac-tabac/tobacco-tabac-strat-2008-eng.htm), or purchased to avoid paying required taxes. Investigators have used an array of questions to estimate this behavior, so we used existing survey items and applied a relatively broad definition of contraband to capture as many smokers open to contraband use as possible, to have a sufficiently large sample to validate the measures. Respondents were classified as having purchased contraband tobacco if they responded “yes” to any of the 5 following questions: “Have you personally ever purchased contraband tobacco,” “In the past six months, have you regularly bought cigarettes outside the US (for US respondents)/in the US (for Canadians), “In the past 12 months, have you bought cigarettes that you think may have been smuggled or stolen,” “Have you EVER purchased cigarettes on an Indian Reservation/from a First Nations Reserve (among non-Indian respondents),” or “Have you EVER purchased cigarettes from a non-retail source, such as out of a person’s home, out a person’s vehicle, or from someone on the street?”

#### Marlowe-crowne social desirability scale

The Marlowe-Crowne Social Desirability Scale (MCSDS) has been used widely to assess social desirability bias among respondents. Because in some instances we were asking about illicit behaviors, respondents were also administered the full 33 item MCSDS to assess possible social desirability bias. Scale scores were classified into three levels: low, medium, high [29]. We hypothesized that those showing high social desirability bias may underreport contraband purchase behaviors and related attitudes.

#### Indicators of contraband tobacco purchase/use

Six scales were adopted from research on attitudes toward non-tobacco contraband products to assess their usefulness for evaluating intended purchase or use of contraband tobacco. The scale items are presented in Table 2 along with the means and standard deviations.^a^ One 5-item scale was specifically adapted to assess attitudes regarding contraband tobacco.

### Analyses

We employed SPSS 21 (IBM, Armonk NY) to assess demographic characteristics, conduct t-tests, and perform exploratory factor analyses (EFA). Dimensionality assessment using EFA involved extraction with Principal Axis Factoring and a Direct Oblimin rotation. Direct Oblimin was selected because it allows for the factors to be intercorrelated. Eigenvalues greater than 1 were accepted as components. Amos (IBM, Armonk, NY) was used to conduct the confirmatory factor analysis (CFA) and structural equation modeling (SEM). Model fit in CFA and SEM was assessed using several metrics, including the comparative fit index (CFI > 0.95), root-mean-square error of approximation (RMSEA < 0.05), goodness of fit index (GFI > 0.95), and minimum discrepancy (CMIN/DF < 3) [30-32]. The SEM used full information maximum-likelihood estimation. The bootstrap resampling technique with bias correction was employed to assess mediation [33]. Convergent and discriminant validity were assessed by examining average variance extracted (AVE > 0.05), maximum shared variance (MSV), and average shared variance (ASV) where MSV < AVE and ASV < AVE indicate validity [34]. The analyses for this article are presented in four parts. First we outline the sample demographics. Second, we performed exploratory and confirmatory factor analyses to assess model fit. Third, we assessed the validity and reliability of proposed scales to assess attitudes toward contraband tobacco. Finally, we tested the structural model for how the measures were related to attitudes toward contraband tobacco.

**Table 2:** **Means, Standard Deviations, and T-Tests of items by contraband tobacco purchase status (N = 483)**

**Scale**		**Non-buyer (60.7%)**	**Buyer (39.3%)**	***T*** **-Test**
**Price quality inference (Lichtenstein et al., 1993** [19]**; Huang et al., 2004** [15]**)** α = 0.880		**Mean (SD)**	**Mean (SD)**	
PQ1	Generally speaking, the higher the price of a product, the higher the quality	2.99 (1.107)	2.81 (1.042)	t(481) = 1.75, p = 0.08
PQ2	The price of a product is a good indicator of its quality	2.89 (1.118)	2.83 (1.040)	t(481) = 0.55, p = 0.58
PQ3	You always have to pay a bit more for the best	2.54 (1.148)	2.39 (1.106)	t(481) = 1.34, p = 0.18
**Risk averseness (Huang et al., 2004** **[** 15 **]** **; Donthu and Garcia, 1999** **[** 36 **]** **)** **α = 0.708**				
RA1	When I buy something, I prefer not taking risks	2.04 (0.869)	2.19 (0.870)	t(481) = -1.88, p = 0.06
RA2	I like to be sure the product is a good one before buying it	1.76 (0.643)	1.86 (0.736)	t(481) = -1.55, p = 0.12
RA3	I don’t like to feel uncertainty when I buy something	1.77 (0.735)	1.97 (0.806)	t(481) = -2.85, p < 0.01
**Subjective norm (Ajzen, 1991** **[** 37 **]** **)** **α = 0.915**				
SN1	My relatives and friends approve my decision to buy counterfeited products	2.20 (1.000)	2.61 (1.077)	t(481) = -4.17, p < 0.00
SN2	My relative and friends think that I should buy counterfeited products	2.09 (0.980)	2.44 (1.096)	t(371.34) = -3.57, p < 0.00
**Behavioral intentions (Zeithaml et al., 1996** **[** 38 **]** **)** **α = 0.939**				
**Considering today, what are the chances that you…**				
BI1	…think about a counterfeited product as a choice when buying something	1.85 (1.054)	2.45 (1.134)	t(382.24) = -5.90, p < 0.00
BI2	…buy a counterfeited product	1.72 (0.950)	2.33 (1.108)	t(359.22) = -6.29, p < 0.00
BI3	…recommend to friends and relatives that they buy a counterfeited product	1.59 (0.904)	2.11 (1.105)	t(346.60) = -5.42, p < 0.00
BI4	…say favorable things about counterfeited products	1.75 (0.970)	2.31 (1.161)	t(352.21) = -5.51, p < 0.00
**Perceived risk (Dowling and Staelin, 1994** [20]**)** α = 0.766				
PR1	The risk that I take when I buy a counterfeited product is high	2.10 (1.052)	2.50 (1.130)	t(382.84) = -3.95, p < 0.00
PR2	There is high probability that the product doesn’t work	1.94 (0.899)	2.32 (1.006)	t(370.79) = -4.29, p < 0.00
PR3	Spending money on a counterfeited product might be a bad decision	1.65 (0.787)	2.03 (0.934)	t(481) = -4.79, p < 0.00
**Integrity (Ang et al., 2001** [21]**) scale measured with PG:** α = 0.866				
INT1	I consider honesty as an important quality for one’s character	1.49 (0.676)	1.75 (0.748)	t(481) = -3.85, p < 0.00
INT2	I consider it very important that people be polite	1.68 (0.735)	1.88 (0.811)	t(481) = -2.80, p < 0.00
INT3	I admire responsible people	1.55 (0.674)	1.82 (0.868)	t(481) = -3.78, p < 0.00
INT4	I like people that have self-control	1.60 (0.679)	1.87 (0.835)	t(481) = -3.91, p < 0.00
**Personal gratification (Ang et al., 2001** [21]**)**				
PG	I always attempt to a have a sense of accomplishment	1.70 (0.734)	2.02 (0.911)	t(481) = -4.15, p < 0.00
**Attitude toward contraband tobacco adapted scale** **α = 0.906**				
ACT1	Considering price, I prefer contraband cigarettes	1.90 (0.876)	2.49 (1.032)	t(356.41) = -6.49, p < 0.00
ACT2	I like shopping for contraband cigarettes	2.04 (1.091)	2.79 (1.241)	t(366.37) = -6.83, p < 0.00
ACT3	Buying contraband cigarettes generally benefits the consumer	2.68 (1.217)	3.24 (1.160)	t(481) = -5.04, p < 0.00
ACT4	There’s nothing wrong with purchasing contraband tobacco	1.72 (0.845)	2.29 (1.115)	t(326.68) = -6.33, p < .0.00
ACT5	Generally speaking, buying contraband cigarettes is a better choice	2.22 (1.117)	2.90 (1.180)	t(481) = -6.37, p < 0.00

Note: All questions asked on a 5-pt. likert scale such that higher scores indicate potential increased preference for CT: SN, INT, PG (1) strongly disagree (5) strongly agree; PQ, RA, PR (1) strongly agree (5) strongly disagree; BI (1) no chance (5) very good chance.

## Results

Overall, nearly 40% of respondents reported purchasing contraband tobacco at some point during the assessed time frames (39.3%). Table 3 outlines the descriptive statistics for the sample population by whether or not the respondent reported purchasing contraband tobacco in the past. Males, (*χ*^2^(1, N = 483) = 17.77, p < .000) and those who identified as Hispanic (*χ*^2^(1, N = 483) = 8.62, p = .003) were more likely to purchase contraband tobacco. Age and ranking on the Marlowe-Crowne Social Desirability Scale were not significantly associated with previous purchase.

**Table 3:** **Inter-correlations between factors**

**Factor**	**ATC**	**PQ**	**INT/PG**	**BI**	**SN**	**PR**
ATC	--					
PQ	−0.024	--				
INT/PG	−0.352	−0.068	--			
BI	−0.599	0.021	0.412	--		
SN	0.524	−0.052	−0.359	−0.489	--	
PR	0.482	0.017	−0.490	−0.536	0.374	--

Means and standard deviations for each of the measures proposed by contraband tobacco purchase status (buyer vs. non-buyer) are presented in Table 1. Overall, those who reported a previous purchase of contraband tobacco were significantly more likely to report higher subjective norms supportive of counterfeit products, higher levels of intentions toward purchasing counterfeit products, lower perceived risk associated with these products, and to have more favorable attitudes toward contraband cigarettes than those who had not purchased contraband tobacco. T-tests showed that the individual measures for price quality and risk averseness were unable to differentiate between buyers and non-buyers of contraband tobacco. The majority of the proposed scales had a high level of internal consistency, with alphas ranging from 0.77 to 0.91. The scale assessing risk averseness had a moderate alpha of 0.70. The risk averseness scale was ultimately dropped from the measurement model due to moderate internal consistency, lack of convergent validity, and invariance issues identified at a later stage of analysis (convergent validity concerns for the latent factor RA: AVE = 0.459, and invariance concerns based on SDS performance).

### Dimensionality

Following this, we employed EFA with principal axis factoring and Direct Oblimin rotation. Dimensionality was present for most of the proposed measures; however, Integrity and Personal Gratification loaded on the same factor, consistent with previous research [16]. The pattern matrix is available in the Additional file 1: Supplemental material. Factor intercorrelations ranged from -0.599 (BI vs ATC) to +0.482 (ATC vs PR), validating the use of oblique rotation (see Table 2).

**Figure 2:**
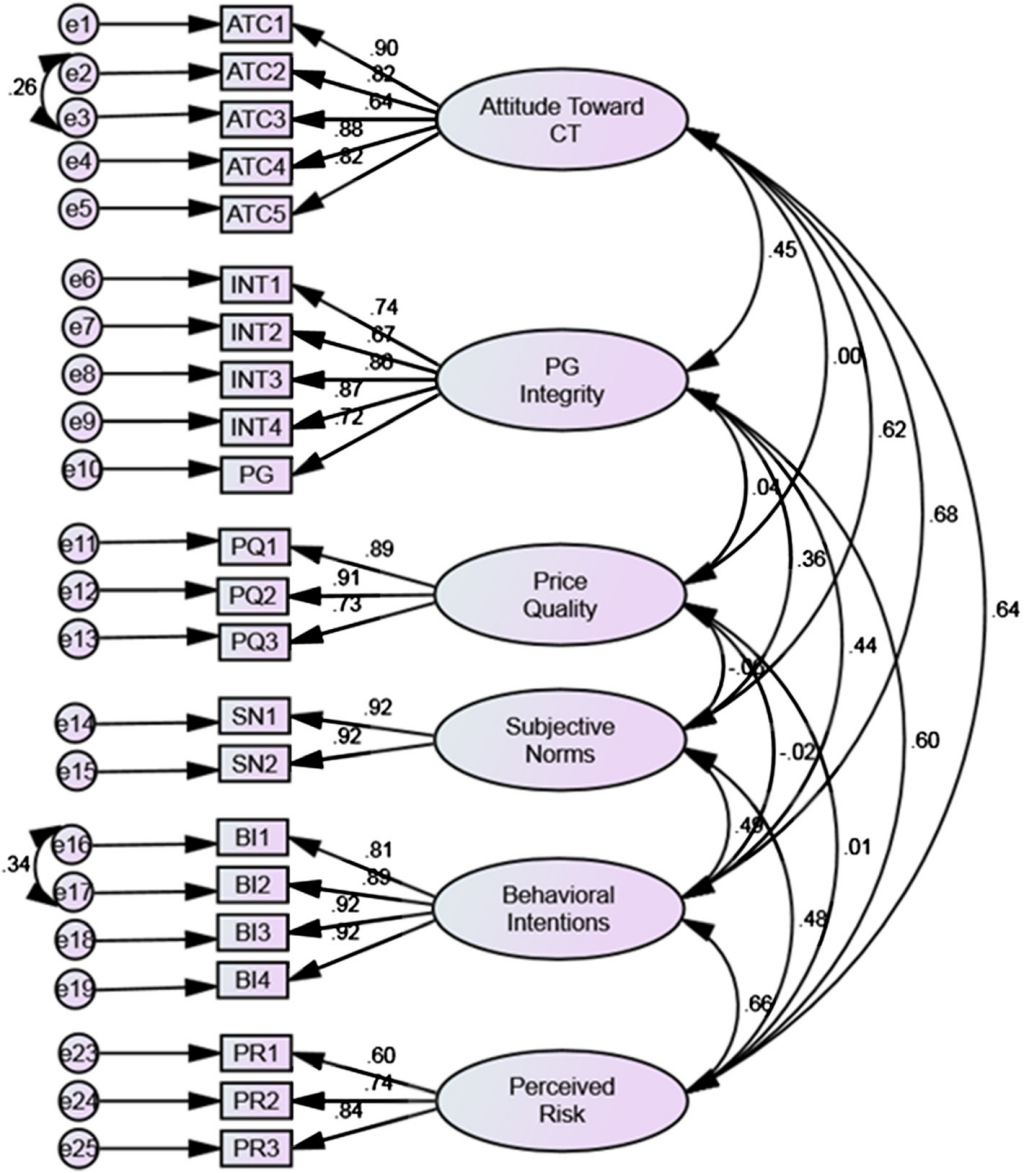
**Final measurement model with regression weights.**

Next, we conducted a CFA on the proposed measurement model (Figure 2). Confirmatory factor analysis demonstrated good model fit (CMIN/DF: 2.145, GFI: 0.925, CFI: 0.970, RMSEA: 0.049, PCLOSE: 0.618), however modification indices and estimated parameter change values indicated the error terms between ATC2 and ATC3 and BI1 and BI2 should be freely estimated. The addition of these improved model fit (CMIN/DF 1.839, GFI: 0.937, CFI: 0.979, RMSEA: 0.042, PCLOSE: 0.978). Invariance tests established that each of the instruments accurately measured the same constructs (or traits) across sex, contraband tobacco purchase status, and performance on the Marlowe-Crowne Social Desirability Scale.

### Reliability and validity of the measurement model

Table 4 presents the validity of the measurement model. The composite reliability (CR) for the measurement model was greater than 0.7, with average variance extracted (AVE) greater than 0.5. Given the CR was greater than the AVE for each measure, convergent validity was established. The AVE was greater than the maximum shared variance (MSV) and the average shared variance (ASV) for each measure, establishing discriminant validity [35].

**Table 4:** **Validity of the measurement model**

**Scales**	**CR**	**AVE**	**MSV**	**ASV**	**BI**	**ACT**	**PGI**	**PQ**	**SN**	**PR**
**BI**	0.936	0.786	0.468	0.270	0.886					
**ACT**	0.909	0.668	0.468	0.292	0.684	0.818				
**PGI**	0.883	0.604	0.364	0.179	0.441	0.449	0.777			
**PQ**	0.886	0.722	0.004	0.001	−0.023	−0.002	0.043	0.850		
**SN**	0.915	0.843	0.383	0.200	0.495	0.619	0.364	−0.062	0.918	
**PR**	0.774	0.537	0.441	0.289	0.664	0.640	0.603	0.007	0.483	0.733

Notes: CR-Composite Reliability; AVE-Average Variance Extracted; Variance; ASV-Average Shared Variance; Square root of AVE (diagonal elements) and correlation between latent variables (off-diagonal elements); Risk Averseness was dropped from the model due to validity issues.

### Structural model

A model evaluating the direct effect for each indicator on behavioral intentions to purchase contraband products was first assessed. The structural model fit the data well (CMIN/DF 1.901, CFI: 0.980, GFI:0.950, RMSEA: 0.043, PCLOSE: 0.907). Lower perceived risk, favorable subjective norms, and having previously purchased contraband tobacco were directly associated with behavioral intentions (see Table 5).

**Table 5:** **Significant relationships for the direct and mediated structural model**

**Hypothesis**	**Direct Effect without Mediator (p-val)**	**Direct Effect with Mediator (p-val)**	**Standardized Indirect Beta (p-val)**	**Mediation observed**
BCT → ATC → BI	0.125 (.001)	0.067 (.110)	.066 (.001)	Full Mediation
SN → ATC → BI	0.219 (.001)	0.063 (.228)	.156 (.001)	Full Mediation
PR → ATC → BI	0.521 (.001)	0.377 (.001)	.153 (.001)	Partial Mediation

**Figure 3:**
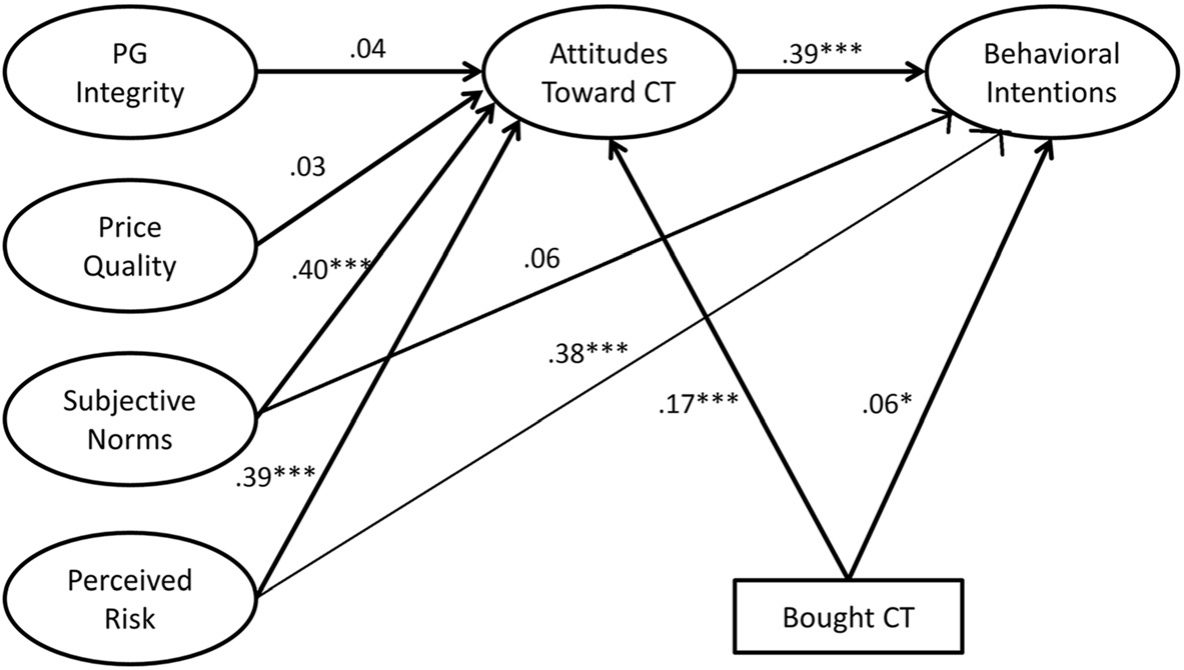
**Final structural model with standardized coefficients.**

Next, a mediation model including the significant direct paths to behavioral intentions and indirect paths from each of the hypothesized indicators through attitudes toward contraband tobacco was tested. The model fit the data well (CMIN/DF:1.879, CFI:0.975, GFI:0.932, RMSEA: 0.043, PCLOSE:0.970). Perceived risk, subjective norms, and prior purchase of contraband tobacco were significantly associated with favorable attitudes toward contraband tobacco which was significantly associated with behavioral intentions in partial support of hypothesis 2, 6, 7, and 8. Bootstrapping was used to assess the significance of the mediated relationships. Perceived risk retained a significant direct effect on behavioral intentions as well as a significant indirect effect through attitudes toward contraband tobacco. Attitudes toward contraband tobacco fully mediated the relationship between subjective norms and behavioral intention and prior purchase and behavioral intention (Table 5). Integrity/Personal Gratification and price quality were not directly associated with attitudes toward contraband tobacco or indirectly with behavioral intentions, not supporting hypothesis 1, 4, or 5(Table 5). Hypothesis 3 was not assessed because the proposed measure was dropped from the model due to validity concerns Figure 3.

## Discussion

There are few validated measures for evaluating attitudes about contraband tobacco, including behavioral intentions. The current research used established measures of non-tobacco contraband/counterfeit attitudes, under the framework of the Theory of Reasoned Action, to assess their relevance for contraband tobacco among a sample of smokers. The structural model indicated that, at least among our sample, indicators of attitude and behavior are partially accounted for by the perceived risks associated with purchasing illicit products. Perceived risk had a strong impact on both attitudes toward contraband tobacco and behavioral intentions. This finding is consistent with economics research showing that increased perceived risk associated with the product quality reduces purchase intentions. Respondents’ perceptions that family and/or friends would support the purchase of these products were also associated with behavioral intentions, though this relationship was fully mediated by attitudes toward contraband tobacco. Overall, the model accounted for over half of the variance in attitudes toward contraband tobacco (53%) and behavioral intentions (54%) to purchase illicit products.

The final model varied from our initial hypotheses, not supporting the hypothesis that price quality and integrity were associated with behavioral intentions or attitudes toward contraband tobacco. While this may be a sample specific finding, it may also suggest that the relationship between antecedents of purchase behavior may be somewhat different than those for non-tobacco contraband. It is possible that the lack of relationship between product quality and attitudes regarding contraband tobacco may be because the majority of contraband tobacco is not counterfeit and is therefore the same product as that commercially sold with appropriate taxation. Product quality may be specifically associated with counterfeit rather than contraband cigarettes, as counterfeit cigarettes are illegally produced by someone other than the trademark holder. The statistical finding was also somewhat expected given the inability for the measure to differentiate between respondents who had previously purchased contraband tobacco and those who had not and is consistent with previous research for non-tobacco contraband/counterfeits [16]. However, it should be noted that, the broad definition of contraband tobacco we applied in the current research may have captured some respondents who purchased counterfeit rather than contraband tobacco products, which may introduce some error.

Attitudes toward contraband tobacco mediated the relationship between prior purchase and behavioral intentions and favorable subjective norms and behavioral intentions. This highlights the importance of prior experience in influencing attitudes, which then influence behavior. Also, the measure of subjective norms was the strongest indicator of attitudes, which highlights the important role that family and friends have in influencing consumer behavior. These findings provide an avenue for public health communications about contraband tobacco to influence attitudes and future behaviors.

### Limitations

This study is subject to a number of limitations. While Amazon Mechanical Turk has been extensively used in research, it by its nature cannot produce a representative sample, so prevalence estimates are not expected to generalize beyond this study; however, the intention of this study was to test the validity of a set of measures not to assess population estimates of contraband tobacco use.

In addition, we employed a very broad definition of contraband tobacco purchase, intending to capture as many smokers as possible who have or would be open to contraband tobacco use. The questions used to capture contraband tobacco use also assessed purchase of these cigarettes across a number of time frames rather than a specified time frame. It is possible that if the questions were phrased differently the results may be somewhat different. Future research should examine the validity of this model with respect to various forms of tax avoidance and evasion, as well as other forms of contraband tobacco purchase.

Finally, this study used and adapted a variety of existing questions from previous research about attitudes and behaviors associated with counterfeit products, though was not exhaustive of the possible social, behavioral, and economic indicators that may be applicable to contraband tobacco. It would be useful to conduct focus groups among smokers to determine how relevant each of the domains is to contraband tobacco attitudes and purchase intentions as well as to determine if other indicators would further our understanding of behavioral intentions.

## Conclusions

Developing a measurement and structural model for understanding attitudes toward contraband tobacco is important given increasing regulation of tobacco products. As taxes on tobacco products increase, among other changes, there may become an increased incentive for consumers to seek out lower price alternatives which may include tapping into illicit markets. Similarly, should regulatory actions establish product standards that significantly alter current products (e.g., removing menthol; reducing nicotine), smokers may be motivated to seek out noncompliant products. Establishing what elements influence attitudes regarding contraband may inform efforts to curtail the development of these markets.

## Endnote

^a^The scale items are presented in Table 2 along with the means and standard deviations.

## Additional file

Additional file 1:
**Supplemental Material: Pattern Matrix.**
