# Supplementary material for: Development of measures assessing attitudes toward contraband tobacco among a web-based sample of smokers
Source: Tob Induc Dis. 2015 Mar 27;13(1):7. doi: 10.1186/s12971-015-0032-0 (PMC4409708; doi:10.1186/s12971-015-0032-0)
Supplement: Additional file 1: — Supplemental Material: Pattern Matrix. [file 12971_2015_32_MOESM1_ESM.docx]

Additional file 1

| **Supplemental Material: Pattern Matrix** | | | | | | |
| --- | --- | --- | --- | --- | --- | --- |
|  | 1 | 2 | 3 | 4 | 5 | 6 |
| ATC2 | .858 |  |  |  |  |  |
| ATC1 | .750 |  |  |  |  |  |
| ATC3 | .739 |  |  |  |  |  |
| ATC4 | .674 |  |  |  |  |  |
| ATC5 | .640 |  |  |  |  |  |
| PQ2 |  | .915 |  |  |  |  |
| PQ1 |  | .895 |  |  |  |  |
| PQ3 |  | .733 |  |  |  |  |
| INT3 |  |  | -.898 |  |  |  |
| INT4 |  |  | -.870 |  |  |  |
| PG |  |  | -.689 |  |  |  |
| INT1 |  |  | -.675 |  |  |  |
| INT2 |  |  | -.674 |  |  |  |
| BI3 |  |  |  | -.925 |  |  |
| BI2 |  |  |  | -.923 |  |  |
| BI4 |  |  |  | -.853 |  |  |
| BI1 |  |  |  | -.786 |  |  |
| SN2 |  |  |  |  | .946 |  |
| SN1 |  |  |  |  | .873 |  |
| PR2 |  |  |  |  |  | .897 |
| PR1 |  |  |  |  |  | .530 |
| PR3 |  |  |  |  |  | .501 |
